# Supplementary material for: Water Intake and Adiposity Outcomes among Overweight and Obese Individuals: A Systematic Review and Meta-Analysis of Randomized Controlled Trials
Source: Nutrients. 2024 Mar 27;16(7):963. doi: 10.3390/nu16070963 (PMC11013432; doi:10.3390/nu16070963)

Table S1. PRISMA Guideline

Section and Topic	Item #	Checklist item	Location where item is reported
**TITLE**			
Title	1	Identify the report as a systematic review.	Page 1
**ABSTRACT**			
Abstract	2	See the PRISMA 2020 for Abstracts checklist.	Page 1
**INTRODUCTION**			
Rationale	3	Describe the rationale for the review in the context of existing knowledge.	Page 1-2
Objectives	4	Provide an explicit statement of the objective(s) or question(s) the review addresses.	Page 1-2
**METHODS**			
Eligibility criteria	5	Specify the inclusion and exclusion criteria for the review and how studies were grouped for the syntheses.	Page 2-3
Information sources	6	Specify all databases, registers, websites, organisations, reference lists and other sources searched or consulted to identify studies. Specify the date when each source was last searched or consulted.	Page 2
Search strategy	7	Present the full search strategies for all databases, registers and websites, including any filters and limits used.	Page 2<br>Table S2
Selection process	8	Specify the methods used to decide whether a study met the inclusion criteria of the review, including how many reviewers screened each record and each report retrieved, whether they worked independently, and if applicable, details of automation tools used in the process.	Page 2-3
Data collection process	9	Specify the methods used to collect data from reports, including how many reviewers collected data from each report, whether they worked independently, any processes for obtaining or confirming data from study investigators, and if applicable, details of automation tools used in the process.	Page 3
Data items	10a	List and define all outcomes for which data were sought. Specify whether all results that were compatible with each outcome domain in each study were sought (e.g. for all measures, time points, analyses), and if not, the methods used to decide which results to collect.	Page 3<br>Table S3

Section and Topic	Item #	Checklist item	Location where item is reported
	10b	List and define all other variables for which data were sought (e.g. participant and intervention characteristics, funding sources). Describe any assumptions made about any missing or unclear information.	Page 3<br>Table S3
Study risk of bias assessment	11	Specify the methods used to assess risk of bias in the included studies, including details of the tool(s) used, how many reviewers assessed each study and whether they worked independently, and if applicable, details of automation tools used in the process.	Page 3
Effect measures	12	Specify for each outcome the effect measure(s) (e.g. risk ratio, mean difference) used in the synthesis or presentation of results.	Page 3
Synthesis methods	13a	Describe the processes used to decide which studies were eligible for each synthesis (e.g. tabulating the study intervention characteristics and comparing against the planned groups for each synthesis (item #5)).	Page 3
	13b	Describe any methods required to prepare the data for presentation or synthesis, such as handling of missing summary statistics, or data conversions.	Page 3-4
	13c	Describe any methods used to tabulate or visually display results of individual studies and syntheses.	Page 3-4
	13d	Describe any methods used to synthesize results and provide a rationale for the choice(s). If meta-analysis was performed, describe the model(s), method(s) to identify the presence and extent of statistical heterogeneity, and software package(s) used.	Page 3-4
	13e	Describe any methods used to explore possible causes of heterogeneity among study results (e.g. subgroup analysis, meta-regression).	Page 4
	13f	Describe any sensitivity analyses conducted to assess robustness of the synthesized results.	Page 4
Reporting bias assessment	14	Describe any methods used to assess risk of bias due to missing results in a synthesis (arising from reporting biases).	Page 3
Certainty assessment	15	Describe any methods used to assess certainty (or confidence) in the body of evidence for an outcome.	Page 4
**RESULTS**			

Section and Topic	Item #	Checklist item	Location where item is reported
Study selection	16a	Describe the results of the search and selection process, from the number of records identified in the search to the number of studies included in the review, ideally using a flow diagram.	Page 4
	16b	Cite studies that might appear to meet the inclusion criteria, but which were excluded, and explain why they were excluded.	Page 3
Study characteristics	17	Cite each included study and present its characteristics.	Page 4
Risk of bias in studies	18	Present assessments of risk of bias for each included study.	Page 4
Results of individual studies	19	For all outcomes, present, for each study: (a) summary statistics for each group (where appropriate) and (b) an effect estimate and its precision (e.g. confidence/credible interval), ideally using structured tables or plots.	Page 5-10
Results of syntheses	20a	For each synthesis, briefly summarise the characteristics and risk of bias among contributing studies.	Page 5-10
	20b	Present results of all statistical syntheses conducted. If meta-analysis was done, present for each the summary estimate and its precision (e.g. confidence/credible interval) and measures of statistical heterogeneity. If comparing groups, describe the direction of the effect.	Page 5-10
	20c	Present results of all investigations of possible causes of heterogeneity among study results.	Page 5-10
	20d	Present results of all sensitivity analyses conducted to assess the robustness of the synthesized results.	Page 9
Reporting biases	21	Present assessments of risk of bias due to missing results (arising from reporting biases) for each synthesis assessed.	Page 4
Certainty of evidence	22	Present assessments of certainty (or confidence) in the body of evidence for each outcome assessed.	Page 11
**DISCUSSION**			
Discussion	23a	Provide a general interpretation of the results in the context of other evidence.	Page 11-12
	23b	Discuss any limitations of the evidence included in the review.	Page 12
	23c	Discuss any limitations of the review processes used.	Page 12
	23d	Discuss implications of the results for practice, policy, and future research.	Page 12-13

Section and Topic	Item #	Checklist item	Location where item is reported
**OTHER INFORMATION**			
Registration and protocol	24a	Provide registration information for the review, including register name and registration number, or state that the review was not registered.	Page 12
	24b	Indicate where the review protocol can be accessed, or state that a protocol was not prepared.	Page 12
	24c	Describe and explain any amendments to information provided at registration or in the protocol.	Page 12
Support	25	Describe sources of financial or non-financial support for the review, and the role of the funders or sponsors in the review.	Page 13
Competing interests	26	Declare any competing interests of review authors.	Page 13
Availability of data, code and other materials	27	Report which of the following are publicly available and where they can be found: template data collection forms; data extracted from included studies; data used for all analyses; analytic code; any other materials used in the review.	Table S3

From: Page, M.J.; McKenzie, J.E.; Bossuyt, P.M.; Boutron, I.; Hoffmann, T.C.; Mulrow, C.D.; Shamseer, L.; Tetzlaff, J.M.; Akl, E.A.; Brennan, S.E.; et al. The PRISMA 2020 statement: An updated guideline for reporting systematic reviews. BMJ 2021, 372, n71. https://doi.org/10.1136/bmj.n71.

Table S2. Search Terms

**PubMed**	(water intake*[tiab] OR water consumption*[tiab] OR water drink*[tiab] OR drinking water[tiab]) AND ("Body Weight"[Mesh] OR weight loss*[tiab] OR weight gain*[tiab] OR weight change*[tiab] OR weight control[tiab] OR “obesity”[Mesh] OR obesity[tiab] OR obese[tiab] OR overweight[tiab] OR body mass index[tiab] OR BMI[tiab] OR waist circumference[tiab] OR body composition[tiab]) NOT (Case Reports[ptyp] OR Comment[ptyp] OR Letter[ptyp] OR Editorial[ptyp]) NOT (“animals”[Mesh] NOT “humans”[Mesh])
**Embase**	(‘water intake’:ti,ab OR ‘water consumption’:ti,ab OR ‘water drink’:ti,ab OR ‘drinking water’:ti,ab) AND (‘body weight'/de OR 'body weight change'/exp OR 'body weight control'/exp OR ‘weight loss*’:ti,ab OR ‘weight gain*’:ti,ab OR ‘weight change*’:ti,ab OR ‘weight control’:ti,ab OR 'obesity'/de OR 'abdominal obesity'/de OR 'adolescent obesity'/de OR 'childhood obesity’/de OR obese:ti,ab OR overweight:ti,ab OR ‘body mass index’:ti,ab OR BMI:ti,ab OR ‘waist circumference’:ti,ab OR ‘body composition’:ti,ab) NOT ('case report'/de OR 'editorial'/de OR 'letter'/de OR 'review'/de) NOT ([animals]/lim NOT [humans]/lim)

Table S3. Main Characteristics of Trials Included

First author<br>Year<br>Country<br>Reference	Trial name<br><br>Participants	Age<br>Male<br>Follow-up period	Intervention<br>vs.<br>Control	Body weight:<br>Mean difference<br>(95% CI*)	BMI:<br>Mean difference<br>(95% CI*)	WC:<br>Mean difference<br>(95% CI*)	Intervention Method	Confounding factors adjusted for
Dennis<br>2010<br>USA<br>(8)	NA<br>overweight or obese (BMI 25-40 kg/m^2)	62.4<br>37.5%<br>12wk	hypocaloric diet +500ml water prior to each daily meal vs. hypocaloric diet only	NA	-0.40<br>(-3.45, 2.65)	-1.20<br>(-8.69, 6.29)	addition	quadratic effect of time
Parretti<br>2015<br>England<br>(19)	NA<br>obese (BMI >30 kg/m^2)	56.5<br>35.7%<br>12wk	500ml water prior to each daily meal vs. imagining stomach was full before meals	-1.20<br>(-2.55, 0.15)	NA	NA	addition	age, sex, ethnicity, deprivation
Peters<br>2014<br>USA<br>(20)	NA<br>overweight or obese (BMI 27-40 kg/m^2)	47.8<br>17.2%<br>12wk	710ml/d water vs. 710ml/d non-nutritive sweetened beverages	1.90<br>(1.03, 2.77)	NA	1.37<br>(-0.00, 2.74)	substitution	
Wong<br>2017<br>USA<br>(22)	NA<br>overweight or obese (BMI >=85th percentile)	14.9<br>28.9%<br>6mo	advice to drink 8 cups of water vs. no advice	0.20<br>(-2.81, 3.21)	-0.20<br>(-1.19, 0.79)	1.00<br>(-2.08, 4.08)	addition	age, sex
Hernández-Cordero<br>2014	NCT01245010	33.3<br>0%	water and education provision vs.	-0.70<br>(1.53, 0.13)	-0.24<br>(-0.59, 0.11)	-0.80<br>(-1.94, 0.34)	substitution	

Mexico<br>(16)	overweight or obese (BMI 25-39 kg/m^2) reporting a sugar sweetened beverages intake of at least 250 kcal/d	6mo	education provision only					
Maersk<br>2012<br>Denmark<br>(18)	NA<br>overweight or obese (BMI 26-40 kg/m^2)	39.0<br>36.0%<br>6mo	water vs. regular cola	-0.70<br>(-3.61, 2.21)	NA	NA	substitution	sex, baseline levels
Madjd<br>2015<br>Iran<br>(17)	IRCT201402177754N5<br>overweight or obese (BMI 27-40 kg/m^2)	31.9<br>0%<br>6mo	substitute water for diet beverages vs. diet beverages	-0.40<br>(-5.02, 4.22)	0.00<br>(-1.65, 1.65)	0.50<br>(-2.59, 3.59)	substitution	
Tate<br>2012<br>USA<br>(21)	choosing healthy options consciously everyday trial<br>overweight or obese (BMI 25-49.9 kg/m^2)	42.0<br>16.0%<br>6mo	water vs. attention control	-4.20<br>(-8.94, 0.54)	NA	-2.80<br>(-6.62, 1.02)	substitution	

Abbreviations: CI, confidence interval; BMI, body mass index; WC, waist circumference; kg, kilogram; m, meter; ml, milliliter; mo, month(s); wk, week(s); NA, not available; d, day.

*Estimated based on provided standard deviation or standard error from respective trials.

Table S4. GRADE Evidence Profile

Certainty assessment							№ of patients		Effect		Certainty
№ of studies	Study design	Risk of bias	Inconsistency	Indirectness	Imprecision	Other considerations	water intervention group	control group	Relative (95% CI)	Absolute (95% CI)	
**Body weight (follow-up: range 3 months to 6 months)**											
7	randomized trials	not serious	serious[a]	not serious	serious[b]	none	465	471	-	WMD **0.33 kg lower** (1.75 lower to 1.08 higher)	⨁⨁◯◯ Low
**BMI (follow-up: range 3 months to 6 months)**											
4	randomized trials	not serious	not serious	not serious	serious[b]	none	192	196	-	WMD **0.23 kg/m^2 lower** (0.55 lower to 0.09 higher)	⨁⨁⨁◯ Moderate
**WC (follow-up: range 3 months to 6 months)**											
6	randomized trials	not serious	not serious	not serious	serious[b]	none	434	443	-	WMD **0.05 cm higher** (1.2 lower to 1.3 higher)	⨁⨁⨁◯ Moderate

CI: confidence interval; **WMD:** weighted mean difference; **BMI:** body mass index; **WC:** waist circumference.

Explanations

a. The WMD’s 95% CI did not overlap across the included trials and high heterogeneity was observed (I^2 > 75 %).

b. The 95% CIs include null effect.

Figure S1: Overview of Risk of Bias Assessment

First author, year	D1	D2	D3	D4	D5	Overall
Dennis, 2010	+	!	!	+	+	!
Hernandez-Cordero, 2014	+	+	+	+	+	+
Madjd, 2015	+	!	!	+	+	!
Maersk, 2012	+	-	!	+	+	-
Parretti, 2015	+	+	+	+	+	+
Peters, 2014	+	+	+	+	+	+
Tate, 2012	+	+	+	+	+	+
Wong, 2017	+	+	+	+	+	+

+	Low risk
!	Some concerns
-	High risk

D1	Randomisation process
D2	Deviations from the intended interventions
D3	Missing outcome data
D4	Measurement of the outcome
D5	Selection of the reported result

Figure S2: Sensitivity Meta-Analysis of Adiposity in Body Weight Comparing Water Intervention vs. Control Groups

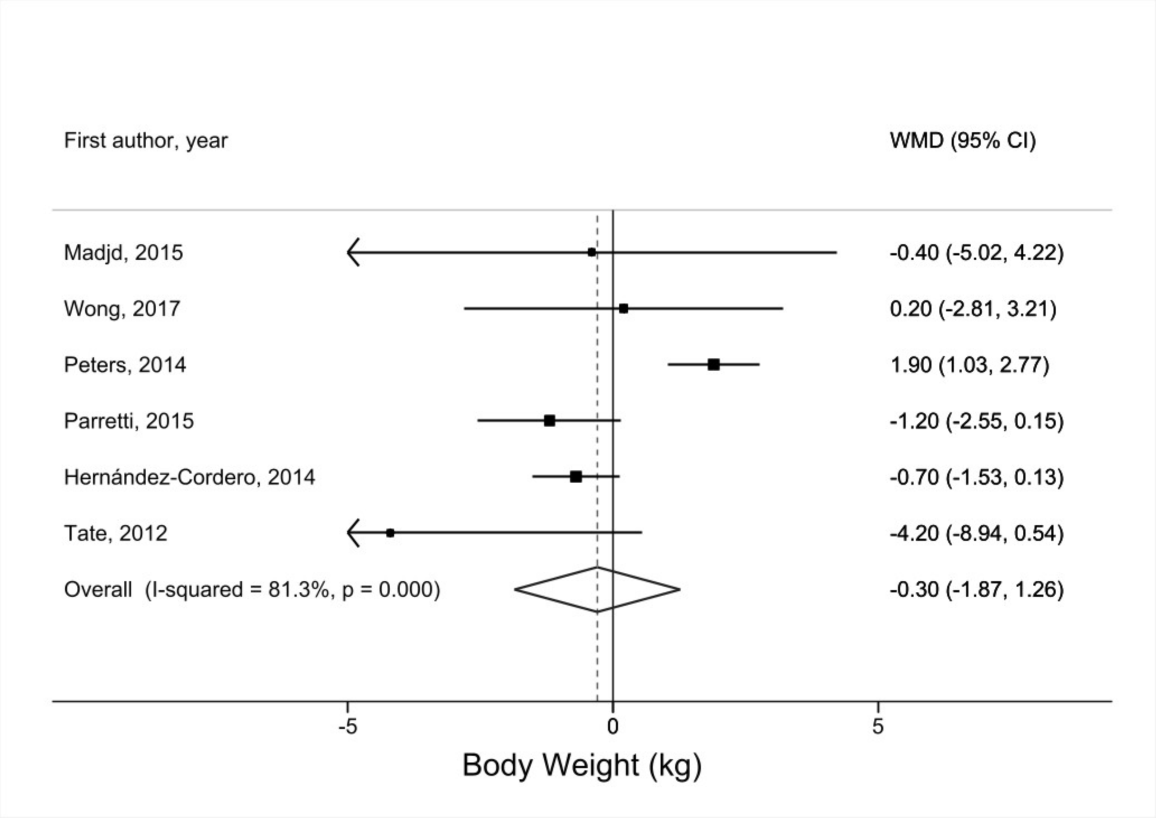

Figure S3: Sensitivity Subgroup Meta-Analysis of Adiposity in Body Weight Comparing Water Intervention vs. Control Groups According to Water Intervention Methods (Adding Water, Substituting Water for Other Beverages)

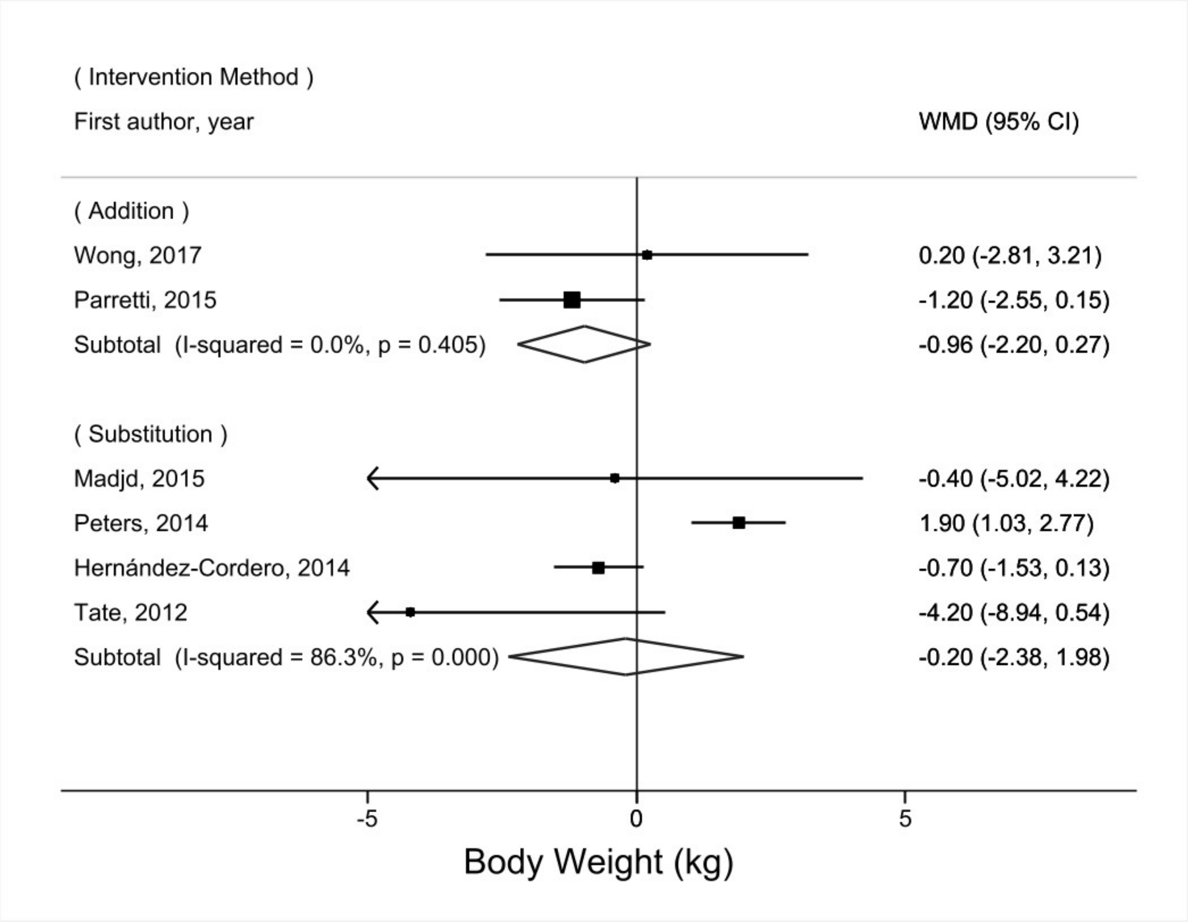

Figure S4: Sensitivity Subgroup Meta-Analysis of Adiposity in Body Weight Comparing Water Intervention vs. Control Groups According to Types of Beverages Replaced (Artificially Sweetened Beverage, Sugar-Sweetened Beverage)

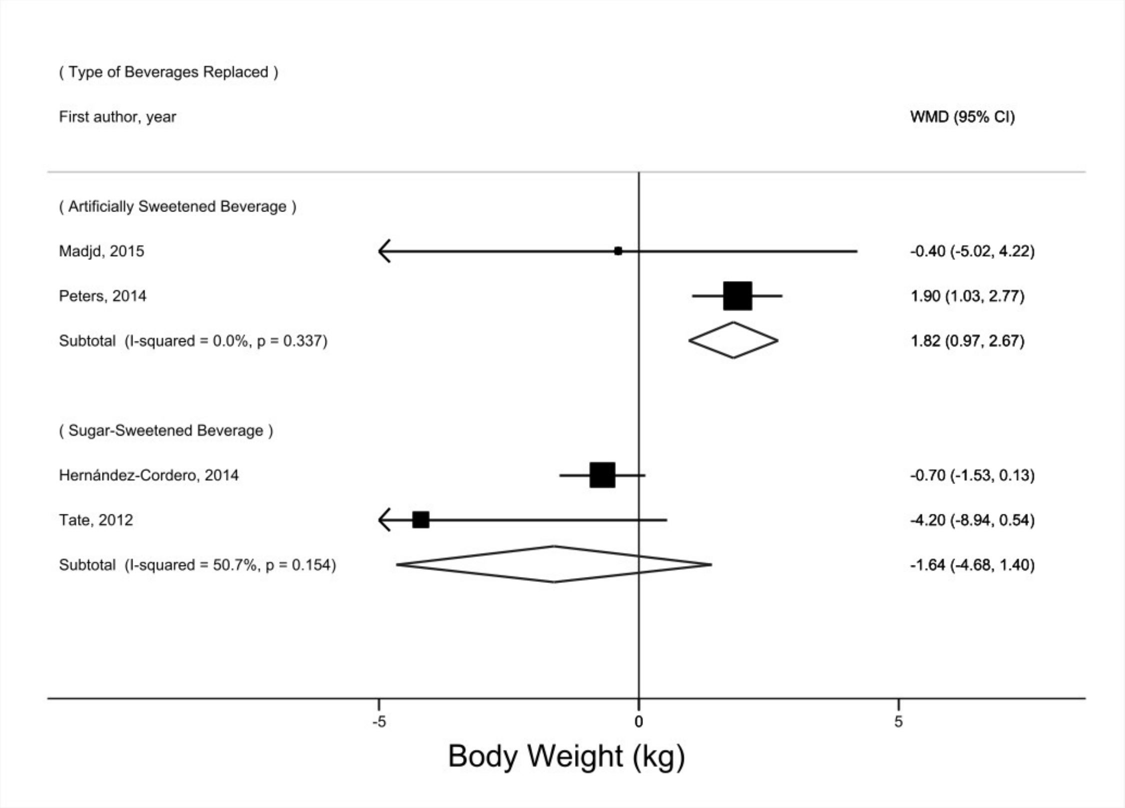

Supplement: Supplementary file 1 [file nutrients-16-00963-s001.zip › nutrients-2893802-supplementary.pdf]
